# Evaluation of Caspase 3 Enzyme and TNF-alpha as Biomarkers in Ureteropelvic Junction Obstruction in Children- a preliminary report

**DOI:** 10.12669/pjms.332.11934

**Published:** 2017

**Authors:** Mehdi Shirazi, Ali Eslahi, Vahidreza Sharifi, Fatemeh Rahimi, Alireza Safarpour

**Affiliations:** 1 Mehdi Shirazi, Department of Urology, School of Medicine, Shiraz University of Medical Sciences, Shiraz, Iran; 2 Ali Eslahi, Department of Urology, School of Medicine, Shiraz University of Medical Sciences, Shiraz, Iran; 3 Vahidreza Sharifi, Urologist, School of Medicine, Shiraz University of Medical Sciences, Shiraz, Iran; 4 Fatemeh Rahimi, Educational Manager, School of Medicine, Shiraz University of Medical Sciences, Shiraz, Iran; 5 Alireza Safarpour, Gastroenterohepatology Research Center, Shiraz University of Medical Sciences, Shiraz, Iran

**Keywords:** Ureteral Obstruction, Urinary Tract Congenital Abnormalities, Tumor Necrosis Factor-α, Caspase 3, Biological Markers

## Abstract

**Objective:**

To determine the applicability of urinary caspase 3 enzyme and TNF-α as biomarkers in children with ureteropelvic junction obstruction (UPJO).

**Methods:**

In this study, 31 unilateral UPJO patients and 33 age- and sex-matched healthy childrens were enrolled. The patients with UPJO consisted of 11 female and 20 male children between the ages of 2 to 62 months old. All participants were evaluated regarding anterior-posterior(AP) diameter and cortical thickness of affected kidney by ultrasonography. Technetium DTPA renal scan and voiding cystourethrogram(to assess vesicoureteral reflux) were performed, pre-operatively. Also, urinary levels of TNF-α and caspase 3 enzyme were checked. Follow-ups included measurement of aforementioned indices in patients: AP diameter and cortical thickness of the affected kidney, as well as TNF-α and caspase 3 levels in urine, three and six months after pyeloplasty.

**Results:**

The results showed highly significant decrease in urinary TNF-α and caspase 3 enzyme (P values < 0.01), approaching the level measured in children without UPJO after six months. Significant decrease in AP diameter and increase in cortical thickness were also noticed (P values < 0.01).

**Conclusion:**

The results of this study strongly support that TNF-α and caspase 3 levels in urine can be used for improvement monitoring in follow-up of UPJO patients after pyeloplasty and can also be potentially used as determining indices for surgical plan but more studies, especially in patients who are not surgical candidates are needed to confirm our observaitons.

## INTRODUCTION

Ureteropelvic junction obstruction (UPJO) is considered as a morbidity of upper urinary tract, with physiologic or structural etiologies, which leads to hydronephrosis of the affected kidney and also is the main cause of congenital obstructive nephropathy.^1^ Manifestations can vary from being asymptomatic to presenting with signs and symptoms like recurrent urinary tract infections, renal stones and pain.^2^ With the help of prenatal screening ultrasonography, hydronephrosis is detected with a prevalence of 0.5% to 1%.^3^,^4^ However, this does not indicate definite presence of obstruction, as only 35% of such cases are afflicted with UPJO.^3^ Indications for surgical intervention include differential function less than 40% which is evaluated by isotopic studies, anterior-posterior (AP) diameter of renal pelvic greater than 20mm, grade III or IV dilatation, pain and infection, and also failure of conservative management leading to more than 10% loss of renal function.^1^ It should be noted that surgical corrections are not always mandatory.^2^ The most important part is preservation of renal function, which for up to 20% of patients requires early interventions, while others can be followed for spontaneous resolution or observation for necessity of treatment at a later time.^3^ Prolonged obstruction eventually leads to progressive loss of nephrons with renal tubular atrophy and interstitial fibrosis.^4^

This process is mediated through pathogenic agents such as reactive oxygen species (ROS), growth factors, nitric oxide, various cytokines and prostaglandins.^5^ Apoptosis of tubular cells along with epithelial-mesenchymal trans-differentiation releases biological markers in the urine.^5^ Thus, it is possible to evaluate the progress of nephropathy by the help of non-invasive study of urinary biomarkers for assessment and prediction of functional and structural changes of kidney and as indicators for necessity of surgical interventions.^3^,^5^ To this date, multiple biomarkers have been reported applicable in diagnosis and long-term follow-up in UPJO; such as, CA-19-9 antigen,^6^ TNF-α, TGF-β,^7^ EGF, endothelin 1, MCP 1 and tubular enzymes.^4^

In the complicated molecular mechanism of apoptosis, Cysteinyl aspartate-specific proteinases (caspases) have a key role, as they lead to the most of morphological and biochemical changes in cellular death. The mechanism of activation of caspases goes through two pathways; receptor mediated death signaling (extrinsic signaling) or mitochondria regulated (intrinsic signaling).^8^ One of the key elements in renal apoptosis is caspase 3.^9^ TNF-α production is increased in response to kidney injuries. TNF-α induces cellular infiltration and fibrin deposition, bringing about reduction of glomerular filtration rate.^10^ Also extrinsic binding of TNF-α to its receptor activate membrane death receptor, that begins the process of apoptosis.^10^

In this study, we aimed to assess the significance of two urinary biomarkers; caspase 3 and TNF-α, in patients with UPJO within six months of follow-up.

## METHODS

Thirty one patients who were referred with diagnosis of asymptomatic unilateral UPJO and 33 normal healthy age- and sex-matched were enrolled. Patients with UPJO consisted of 11 female and 20 male children between the ages of 2 to 62 months old. Children were selected among patients referred to Namazi Hospital, Shiraz, Iran and were candidates for surgical treatment of UPJO.

All procedures performed in studies involving human participants were in accordance with the ethical standards of the institutional and national research committee and with the 1964 Helsinki declaration and its later amendments or comparable ethical standards. Informed consent was obtained from all individual participants included in the study.

Healthy group consisted of 12 female and 21 male children those referred to a general pediatric outpatient clinic, where all were periodically monitored for their development and growth. This study was performed from September 2012 to January 2015.

Criteria for pyeloplasty consisted of differential function less than 40% which is evaluated by isotopic studies, AP diameter of renal pelvis greater than 20mm and also failure of conservative management leading to more than 10% loss of renal function.

Exclusion criteria included symptomatic patients (such as UTI or pain), bilateral UPJO, presence of urinary tract stone, presence of vesicoureteral reflux (VUR), chronic renal failure (CRF), malignancy, abnormal liver function, and abnormal level of creatinine and blood urea nitrogen and also patients with fever or any viral infections. Parents were required to fill out a written consent Forms after they were thoroughly informed on the process of the study.

Before surgery, all participants were evaluated regarding AP diameter and cortical thickness of affected kidney by ultrasonography, which were all done by the same radiologist, ^99^technetium DTPA renal scan and voiding cystourethrogram were performed (to assess VUR). Also, urinary levels of TNF-α and caspase 3 enzyme were checked in fresh first morning voided urine samples by the use of a commercial enzyme-linked immune absorbent assay kit (RayBiotech, Norcross, GA) and human cysteinyl aspartated specific proteinases 3 (CASPASE-3) ELISA kit (Hangzhou Estabiopharm CO), respectively. Healthy children were assessed only for TNF-α and Caspase 3 in order to obtain a mean level of these factors for comparison.

Subsequently, patients underwent surgery, which were all done by the same pediatric urologist. Follow-ups included measurement of aforementioned indices: AP diameter and cortical thickness of affected kidney, as well as TNF-α and CASPASE 3 enzyme levels in urine, three and six months after pyeloplasty.

Data were analyzed with repeated measure test using SPSS 19 software. P value < 0.05 was considered statistically significant.

## RESULTS

This study included 31 patients with UPJO (mean age ± SD = 26.44 ± 18.93 months) and 33 healthy children (mean age =29.79 ± 20.52 months. Renal function was measured pre-operatively in patients. The results showed a maximum function of 37% and a minimum of 23% (Mean ± SD = 30.17 ± 3.47).

TNF-α and caspase 3 enzyme levels were checked in healthy children, in order to have a basis for comparison with the patients group. Mean values were 12.75 (5-28)pg/mg creatinine and 7.91 (6-13)ng/mg creatinine for TNF-α and caspase 3 enzyme, respectively.

As regards the AP diameter of renal pelvis, measured in ultrasonography, the results showed significant reduction during the six months follow-up (P value<0.01). The subsequent mean±SD APDs were 37.37±7.45 mm (pre-operatively), 28.86±6.81 mm (after 3 months) and 14.65±4.55 mm (after 6 months). Furthermore, analysis of data for cortical thickness (mm) was supportive of significant increase (P value<0.01). The lowest thickness was detected pre-operatively (4.20±1.23 mm), while this value increased to 9.17±2.3 mm six months after the surgery. The results of ultrasonography evaluation are shown in Table-I. Also, p values for all pairwise comparisons were less than 0.01.

**Table-I:** Imaging data of patients: AP diameter and cortical thickness.

*Variables*		*Pre-operatively*	*After 3 Months*	*After 6 Months*	*P value*
AP diameter (mm)	Mean±SD	37.37±7.45	28.86±6.81	14.65±4.55	<0.01
	Min – Max	27 - 51	18 – 45	10 - 28
Cortical Thickness (mm)	Mean±SD	4.20±1.23	6.51±2.08	9.17±2.34	<0.01
	Min – Max	2 - 7	3 – 11	5 - 15

The two biochemical markers measured in this study showed a significant reduction. Both factors showed a slightly faster decrease in the first three months. All P values, repeated measure test and pairwise comparisons, were less than 0.01. (Table-II)

**Table-II:** Urinary TNF-alpha and caspase 3 enzyme levels during the 6 months follow-up.

*Variables*		*Pre-operatively*	*After 3 Months*	*After 6 Months*	*P value*
TNF-alpha (pg/mg creatinine)	Mean±SD	80.00±26.07	32.89±7.77	13.34±6.02	<0.01
	Min Max	35 - 125	25 – 52	6 – 29
Caspase 3(ng/mg creatinine)	Mean±SD	22.38±4.34	13.90±4.56	8.34±2.13	<0.01
	Min Max	15 – 32	8 – 24	7 – 13

The amount of TNF-alpha and Caspase 3 enzyme were compared in children with UPJO and the healthy group pre-operatively and six months after the operation. The results are seen in Table-III.

**Table-III:** Comparison of TNF-alpha and Caspase 3 enzyme in patients and control group pre-operatively and after six months.

*Biomarkers*	*Patients*	*Control*	*P value*
TNF-alpha Pre-operatively	80±26.07	13.90±5.3	<0.001
TNF-alpha After six months	13.34±6.02	13.70±5.80	0.80
Caspase 3 Pre-operatively	3.14±0.61	1.19±0.25	<0.001
Caspase 3 After six months	1.17±0.30	1.19±0.25	0.41

Both urinary biomarkers, TNF-alpha and Caspase 3, were measured in patients and control group pre-operatively with significant statistic difference (P-value<0.001, P-value<0.001 respectively). But after six months there was no significant difference in TNF-alpha and Caspase 3 between the two mentioned groups (P-value=0.8, P-value=0.4 respectively).

## DISCUSSION

Indications for surgical treatment of UPJO, an obstructive abnormality affecting the urinary tract with multiple complications, remain controversial despite various imaging and laboratory studies. In this survey, we aimed to evaluate the applicability of two biomarkers, TNF-α and caspase 3 enzyme, in order to provide practitioners with another tool for decision making and follow up. In our study imaging results, indicating significant improvements after three and six months, were in accordance with the decreasing of urinary TNF-α and caspase 3 enzyme in patients. In both follow-ups, these biomarkers showed significant decrease, however, the rate of decrease was faster in the first three months interval. These changes were compatible with the structural changes detected in ultrasonography, which were in favor of significant improvement of the obstructive hydronephorsis.

In patients, urinary TNF-α and caspase 3 enzyme levels were similar to healthy patients after six months. This fact also reveals achieving decreased levels can be expected six months after pyeloplasty. Combined use of ultrasonography findings and urinary level of TNF-α and caspase 3 enzyme as biomarkers can help better evaluate the improvement of patients.

Previous literature has already shed light on the role of TNF-α as a mediator in obstructive-induced renal injury,^11^ which can be produced by renal cortical tubular cells independent of inflammatory cell infiltration.^12^ The role of TNF-α in obstructive renal cell apoptosis was studied by Misseri et al in rats.^13^ The authors reported increased TNF-α level and production induced by obstruction, declaring that this factor is responsible for apoptotic renal cell death and its neutralization can be considered as a potential therapy of obstructive renal injury.^13^ This finding has been reported in similar studies as well.^14^,^15^ In an experimental study by Madsen et al., which investigated the urine and kidney cytokine profiles of hydronephrosis in rats, TNF-α was reported to have significantly increased concentration in inner medulla and urine.^16^ Moreover, evaluation of male, female, castrated male, and testosterone-treated oophorectomized female rats three days after unilateral ureteral closure, demonstrated a significant increase of TNF-α production, caspase activity, apoptotic cell death, tubulointerstitial fibrosis, and renal dysfunction in male and testosterone-treated oophorectomized female. These findings demonstrate that testosterone may also play role in TNF production and renal dysfunction after renal obstruction.^17^

Additionally, it has been shown that higher level of TNF-α is significantly correlated to abnormal ultrasound results in children with acute pyelonephritis.^18^ Unfortunately, studies on human subjects and in regards to monitoring of TNF-α level with follow-ups are scarce.

Caspases are cysteine proteases, among which caspase 3 enzyme is considered as one of the markers of cell death in tubular cell apoptosis.^19^,^20^ Both intrinsic and extrinsic pathways can trigger activation of caspase 3.^21^ Activation of caspase through cell death receptors is regulated by a subset of the TNF receptor superfamily, which includes Fas/CD95, TNF receptor (TNFR)-1, and death receptor-3.^8^ Release of cytochrome c from mitochondria through stress stimuli, induces activation of caspase 9, which in turn leads to activation of caspase 3 enzyme. Subsequently, caspase-activated DNase leads to internucleosomal DNA fragmentation, a hallmark of apotosis.^21^,^22^ Besides this genetical degradation of cell, caspases cleave a large number of protein substrates in late stages of apotosis, which alters cellular morphology. This structural disaggregation via caspase 3 has been recorded to occur in renal cells.^23^

Caspase 3 has been reported to be activated in stretch-induced renal cell apoptosis in obstructive nephropathies.^24^ Inhibition of caspase activity decreases tubular cell apoptosis in renal ischemia-reperfusion injuries to kidney, therefore preventing inflammation and fibrosis.^25^ To our knowledge this study is the first report that assess the concentration of caspase 3 in urine of children with UPJO.

Evaluation of the two biomarkers, which were studied in this paper, has been performed on patients who were candidates for surgical treatment. It is suggested that more studies could clarify if the rise of TNF-α or caspase 3 enzyme can be used as indices for surgical intervention in patients under conservative treatment. The results can be more reliable if larger numbers of patients are involved.

## CONCLUSION

The results of this study strongly support that TNF-α and caspase 3 enzyme levels in urine can be used for improvement monitoring in follow-up of UPJO patients after pyeloplasty. It can also can potentially be used as determining indices for surgical plan, but to achieve this goal, more studies, especially in patients who are not surgical candidate, needs to be performed. The normal levels of these biomarkers can be expected to be achieved after six months.
